# Importance of differential diagnosis of EBV mucocutaneous ulcer and EBV-positive diffuse large B-cell lymphoma: A case report

**DOI:** 10.1097/MD.0000000000037243

**Published:** 2024-02-23

**Authors:** Yoo Ree Hong, Jeong-Seung Kwon, Hyung-Joon Ahn, Seung-Yong Han, Eunae Cho, Bok Eum Kim

**Affiliations:** a Department of Orofacial Pain and Oral Medicine, Sun Dental Hospital, Yonsei University College of Dentistry, Seoul, Korea; b Department of Orofacial Pain and Oral Medicine, Dental Hospital, College of Dentistry, Yonsei University College of Dentistry, Seoul, Korea; c Department of Oral Pathology, Dental Hospital, College of Dentistry, Yonsei University College of Dentistry, Seoul, Korea; d Department of Advanced General Dentistry, Dental Hospital, College of Dentistry, Yonsei University College of Dentistry, Seoul, Korea.

**Keywords:** Epstein-Barr virus infections, lymphoma, large B-cell, diffuse, neoplasms, oral ulcer, virus diseases

## Abstract

**Rationale:**

Epstein-Barr virus mucocutaneous ulcers (EBVMCUs) were officially recognized as a clinicopathologic entity in the 2017 revision of the World Health Organization classification, which often occurs in the elderly or in immunosuppressive condition presented as an isolated ulcerative lesion. EBVMCUs are defined as “shallow, sharply circumscribed, mucosal or cutaneous ulcers with underlying polymorphous infiltration.” It mostly involves oral mucosa, but some appear in skin or gastrointestinal tract. Typically, patients with EBVMCUs display a slow disease progression and may even undergo spontaneous regression.

**Patient concerns:**

This report describes the case of a 76-year-old woman who visited our outpatient clinic with the chief complaint of inflammation and ulceration on lower labial, lower right lingual gingiva seemed like acute necrotizing ulcerative gingivitis, and malignancy.

**Diagnoses:**

She was diagnosed with EBVMCU after tissue biopsy.

**Interventions:**

Since most oral ulcerations usually appear in nonspecific form, it is important to check thoroughly for any underlying immunosuppressive systemic conditions and laboratory test results in case of viral infection. But she has no remarkable underlying immunosuppressive disorder.

**Outcomes:**

For this patient, she was initially diagnosed with EBVMCU and showed spontaneous healing, but then relapsed after 4 to 6 months. The patient was re-diagnosed as EBV-positive diffuse large B-cell lymphoma (EBV-positive DLBCLs) after re-biopsy.

**Lessons:**

EBVMCU shows similar symptoms to malignant lesions or acute necrotizing ulcerative gingivitis but shows spontaneous healing. However, in case of EBV-positive DLBCLs, failing to detect and treat the disease in its early stages can lead to a fatal outcome. Thus, this case report highlights the differential diagnosis and appropriate treatment of EBVMCU and EBV-positive DLBCLs.

## 1. Introduction

Epstein-Barr virus (EBV) is a common type of human herpes virus (human herpes virus 4) that can lead to lymphomas and lymphoproliferative disorders like EBV-positive diffuse large B-cell lymphomas (EBV-positive DLBCLs) in severe cases. Individuals on immunosuppressive drugs,^[1,2]^ organ transplant recipients,^[3,4]^ or those with immune deficiencies, face a higher risk of EBV-associated lymphoproliferative disorders (EBV-LPDs). However, even non-immunosuppressed elderly are at risk due to age-related immunosenescence.^[5]^

Epstein-Barr virus mucocutaneous ulcers (EBVMCUs) are presented as a solitary, well-circumscribed isolated ulcerative lesion. Reported cases showed self-limiting course when immunosuppressive agents were discontinued, which is different from EBV-associated lymphomas.

This paper presents the case who complained of a persistent ulceration on the lower gingiva. Despite clinical resemblance to malignancy or acute necrotizing ulcerative gingivitis, the biopsy confirmed as EBVMCUs. Re-biopsy was done after symptom recurrence and the diagnosis was revised to EBV-positive DLBCLs.

This study received approval from the Institutional Review Board of Yonsei University Dental Hospital (IRB approval number: 2-2022-0074). The IRB waived the need to obtain informed consent given the retrospective nature of the study.

## 2. Case description

**Figure 1.**
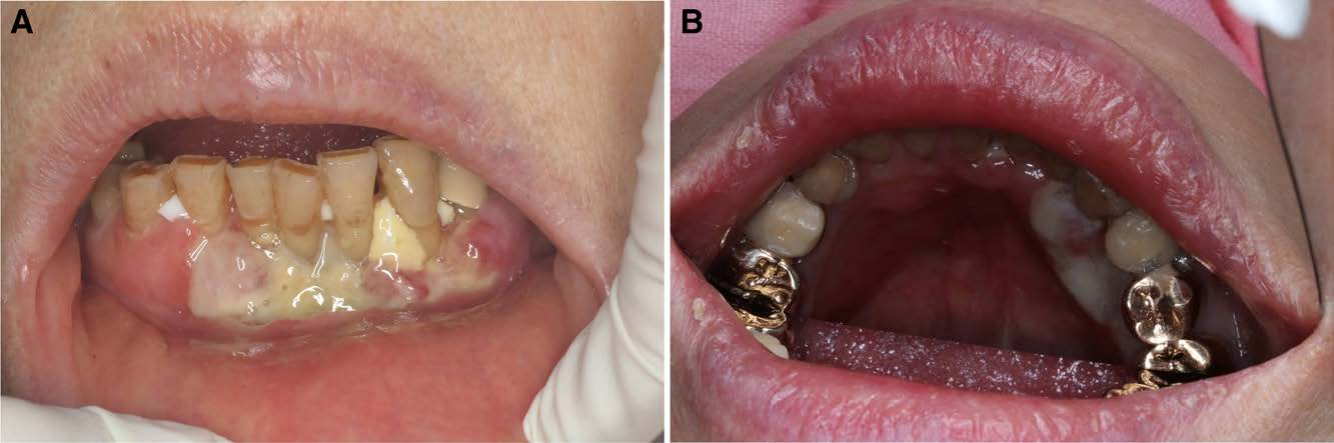
Clinical photo taken in first visit. (A) Lower labial gingiva and (B) lower lingual gingiva.

A 76-year-old woman was referred to Department of Orofacial Pain and Oral Medicine of Yonsei University Dental Hospital (Seoul, Korea), due to lower labial gingiva ulcer (#32-43 area) present for 50 days (Fig. 1). Tissue biopsy at prior hospital revealed EBVMCU, but she had no remarkable immunosuppressive conditions.

During her initial visit, the patient complained of aching and lancinating pain (Numeric Rating Scale 6) in her lower anterior labial/lingual gingiva worsened by eating and tooth brushing. Clinically, the ulcer on the lower labial gingiva raised concerns of malignancy. Severe gingival recession and poor oral hygiene were also observed. She was prescribed Fullgram® (clindamycin), Tylenol® (acetaminophen), and Hexamedine® (chlorhexidine) gargle for 1 week with a preliminary diagnosis of oral candidiasis, suspected necrotizing gingivitis, and potential malignancy.

A whitish-yellowish ulcerative lesion showed tenderness, redness, and swelling but no bleeding. The patient received medications including Methylon® (methylprednisolone), prednisolone-ampicillin gargle. Further evaluation included laboratory tests like C-reactive protein, herpes simplex virus, and varicella-zoster virus.

**Figure 2.**
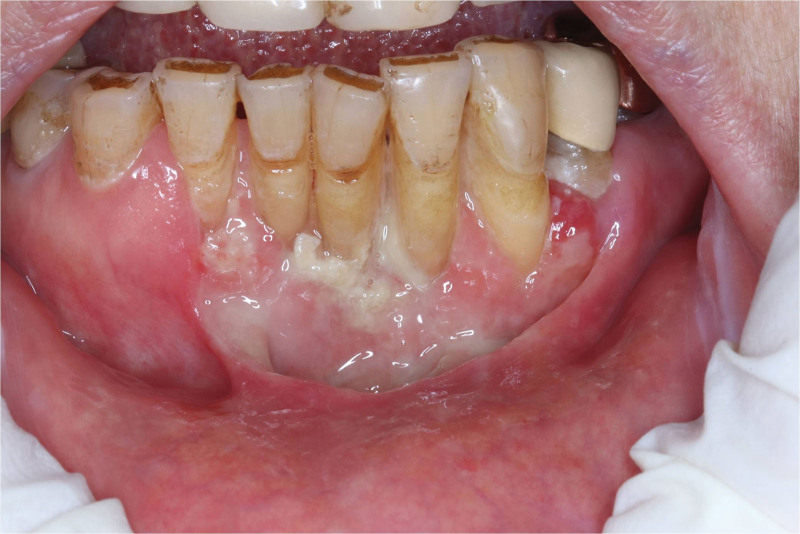
Clinical photo taken after use of systemic corticosteroids with topical fluconazole.

Two weeks later, the pain in the lower labial gingiva had lessened to a mild level (Numeric Rating Scale 2–3). The ulcerative lesion had partially healed (Fig. 2), and there were whitish patches and plaques on both buccal mucosae, implying oral candidiasis. Laboratory results showed an elevated C-reactive protein level of 11.6 (normal range 0–8), while other tests were unremarkable. An enhanced magnetic resonance imaging of the face detected only slight enhancement in the anterior mandible. The patient was prescribed Methylon® once more, along with topical fluconazole (Diflucan®).

Medications showed some improvement, but the lesion persisted. Laboratory tests showed elevated levels of liver function markers, blood urea nitrogen, and creatinine, so systemic corticosteroids were replaced with topical treatment. To gain more insights, the biopsy slide was sent to the oral pathology department at Yonsei University Dental Hospital for reevaluation, which once again confirmed EBVMCU.

**Figure 3.**
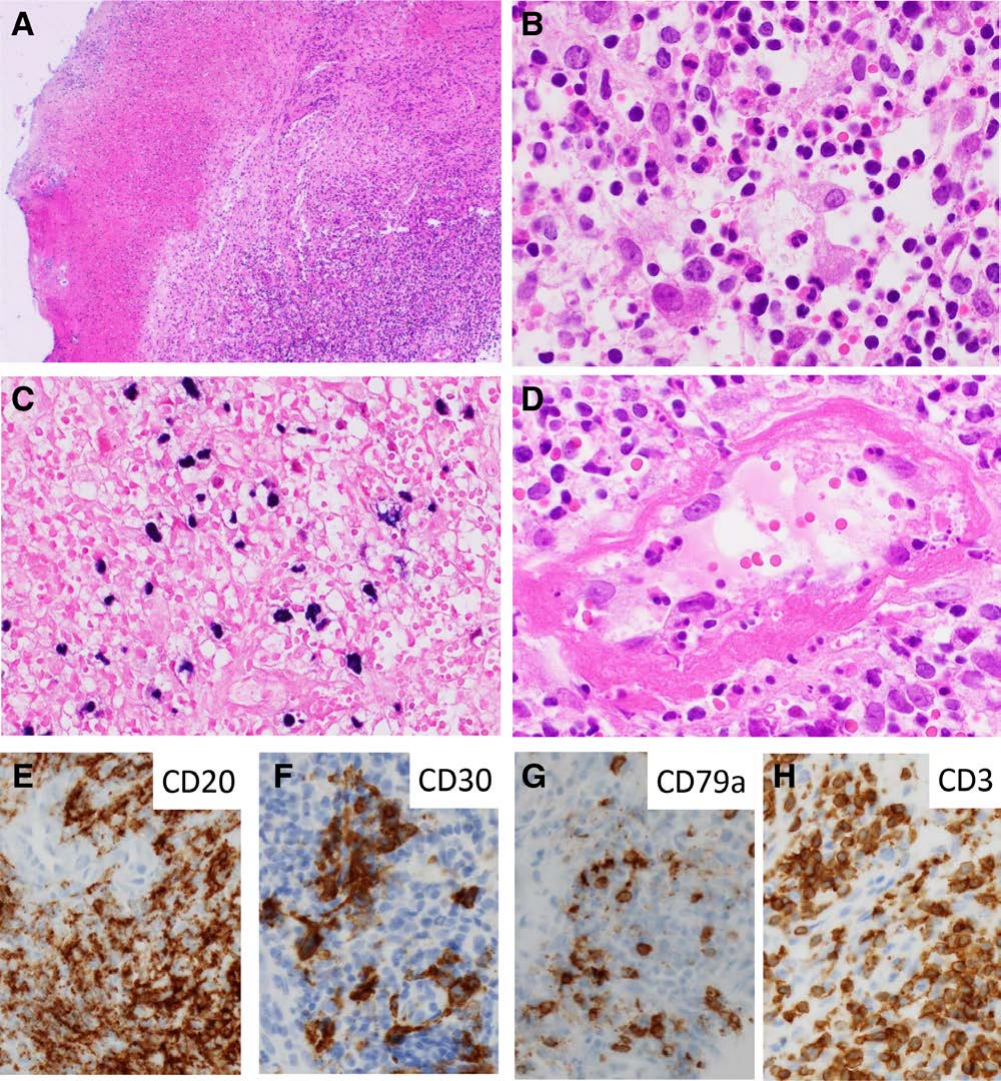
Histopathologic examination and molecular pathologic results. (A) Dense inflammatory cell infiltrates in superficial submucosa on mucosal ulcer (hematoxylin and eosin, ×40). (B) More abundant larger transformed cells with basophilic, prominent nucleoli along with histiocytes and neutrophils (hematoxylin and eosin, ×400). (C) Large cells positive for Epstein-Barr encoded RNA (EBER), as detected through in situ hybridization (×200). (D) Angioinvasion by variable inflammatory cells (hematoxylin and eosin, ×400). CD20 (E), CD30 (F), and CD79a (G) positive large cells (immunohistochemical staining, ×200). (H) CD3+ reactive T cells infiltration (×200, immunohistochemical staining).

Histopathologic examination revealed the ulcerative lesion with the inflammatory cells infiltrates, composed of small-sized to large-sized lymphocytes, histiocytes, and neutrophils (Fig. 3A). The scattered large-sized lymphocytic cells showed atypia with basophilic, prominent nucleoli (Fig. 3B), and they were positive for Epstein-Barr encoding region by in situ hybridization (Fig. 3C). Angioinvasion of the lesional cells was observed (Fig. 3D). Immunohistochemical staining revealed that the large-sized lymphocytic cells were CD20 (+), CD30 (+), and CD79a (+) (Fig. 3E–G). Immunohistochemical staining for PAX5 was negative, presumably due to tissue necrosis. CD3(+) reactive T cells infiltrated within the lesion (Fig. 3H). Considering histopathologic examination and immunohistochemical results, the diagnosis was established as EBVMCUs.

**Figure 4.**
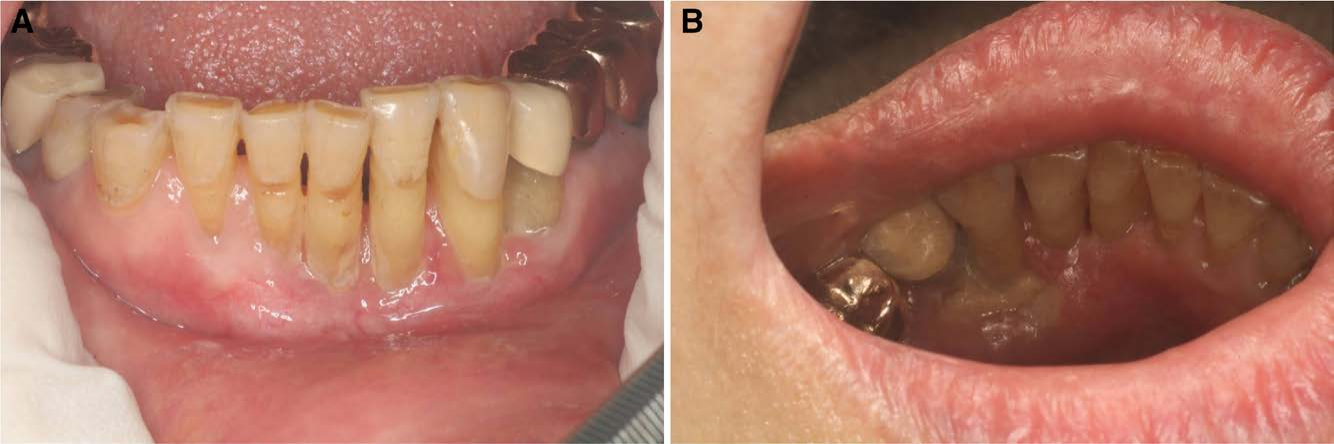
Clinical photo taken 4 months after onset. (A) Lower labial gingiva and (B) Lower lingual gingiva.

Two months after her initial visit (4 months from symptom onset), the lesion had significantly improved (Fig. 4). She had undergone thorough evaluations in other hospital (including divisions of infectious disease, pulmonology, and gastroenterology) where biopsy was done, and no remarkable findings were reported. After an additional month, the lesion had almost completely resolved, so steroid treatment was discontinued.

**Figure 5.**
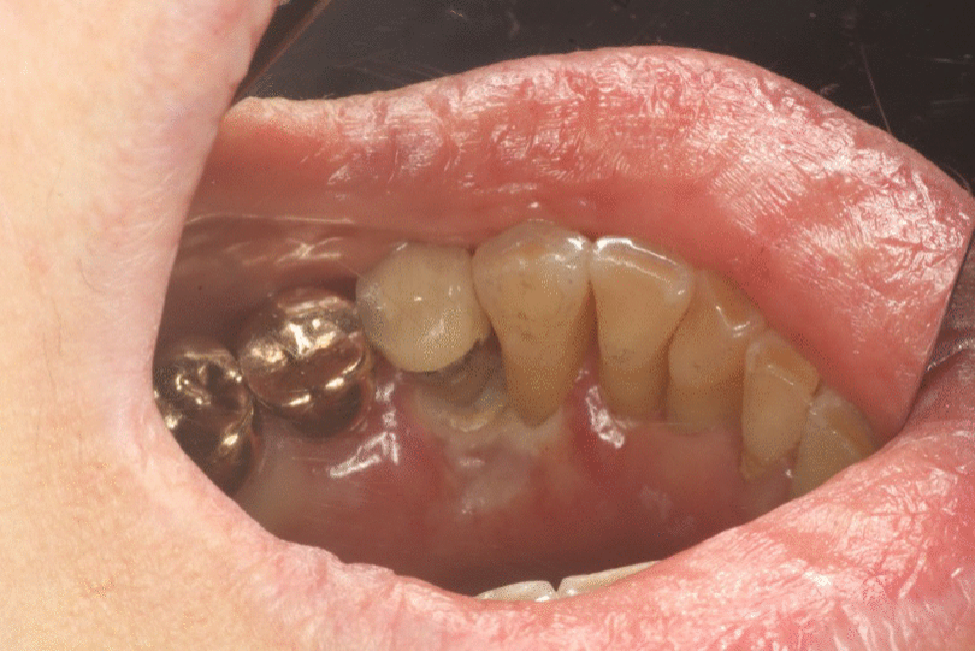
Clinical photo of recurrence 4 to 6 months after spontaneous healing.

The lesion improved without additional medication, but 4 to 6 months later, it recurred on #33 and #34 lingual gingiva (Fig. 5). Re-biopsy and laboratory tests were recommended, but the patient declined. Two months after the recurrence, her symptom worsened, and she developed redness and swelling in the chin area. Re-biopsy was eventually performed, leading to the diagnosis of EBV-positive DLBCLs. She began chemotherapy and radiation treatment in the Department of Hematology and Infectious Diseases, but unfortunately, her oral lesion and overall condition did not improve. She passed away despite ongoing treatment.

## 3. Discussion

EBV often infects young adults and is frequently asymptomatic. Accurate diagnosis and a multidisciplinary approach are crucial as EBV can be linked to severe conditions like nasopharyngeal carcinoma, Burkitt, and some other lymphomas.^[6]^ The treatment strategy includes conservative care (discontinuation of immunosuppressive drugs and follow-up) or chemotherapy or radiation therapy.^[7]^

EBVMCU is distinct from EBV-associated lymphomas because it leads to localized lesions and carries a favorable prognosis. While its clinical presentation may mimic malignancy, discontinuing immunosuppressive medications can often lead to remission,^[5]^ highlighting the risk of overtreatment. To ensure accurate diagnosis and management, laboratory tests, biopsies, and supportive care are essential. Consultation with a hematology department is crucial, particularly when differentiating it from EBV-DLBCLs.

In this case of EBVMCU, the suspected cause is the patient’s periodontal condition. The patient had severe gingival recession with deep periodontal pockets and loose teeth. It is plausible that the chronic periodontal lesion provided a susceptible site for EBVMCU. There is a precedent in a case report linking periodontal lesions to EBV secretion in saliva in *Pemphigus vulgaris* patient.^[8]^ Further research is needed to investigate the development of EBVMCU from periodontal disease.

Recently, there have been reports of EBV-DLBCLs originating from EBVMCU.^[9,10]^ A study in Japan emphasized the challenge of EBVMCU and EBV-DLBCL differential diagnosis based solely on pathological and genetic findings. It underscores the importance of collecting comprehensive clinical information, including medical history, lesion location, and laboratory data, for an accurate differential diagnosis.^[11]^

In this patient’s case, the initial diagnosis was EBVMCU, and subsequent re-biopsy finally revealed EBV-positive DLBCL after recurrence. The median survival rate for EBV-positive DLBCL, is known to be <2 years, and this patient also passed away 1 year and 7 months after the onset. While EBV-positive DLBCL often occurs alongside underlying immunosuppressive or systemic diseases, this patient had no significant systemic conditions. As previously mentioned, distinguishing between EBVMCU and EBV-positive DLBCL based solely on pathological findings can be challenging. Therefore, it is essential to continue outpatient follow-up and conduct a comprehensive systemic evaluation, even if EBVMCU is initially diagnosed and exhibits signs of spontaneous healing.

## Author contributions

**Conceptualization:** Yoo Ree Hong, Jeong-Seung Kwon, Hyung-Joon Ahn, Bok Eum Kim.

**Data curation:** Seung-Yong Han, Eunae Cho.

**Formal analysis:** Yoo Ree Hong, Jeong-Seung Kwon, Hyung-Joon Ahn, Bok Eum Kim.

**Writing – original draft:** Yoo Ree Hong.

**Writing – review & editing:** Yoo Ree Hong, Jeong-Seung Kwon, Hyung-Joon Ahn, Seung-Yong Han, Eunae Cho, Bok Eum Kim.
